# Molecular and Functional Characterization of Inhibitor of Apoptosis Proteins (IAP, BIRP) in *Echinococcus granulosus*

**DOI:** 10.3389/fmicb.2020.00729

**Published:** 2020-04-22

**Authors:** Jiafei Zhan, Hongyu Song, Ning Wang, Cheng Guo, Nengxing Shen, Ruiqi Hua, Yuan Shi, Christiana Angel, Xiaobin Gu, Yue Xie, Weimin Lai, Xuerong Peng, Guangyou Yang

**Affiliations:** ^1^Department of Parasitology, College of Veterinary Medicine, Sichuan Agricultural University, Chengdu, China; ^2^Department of Veterinary Parasitology, Faculty of Veterinary Sciences, Shaheed Benazir Bhutto University of Veterinary and Animal Sciences, Sakrand, Pakistan; ^3^Department of Chemistry, College of Life and Basic Science, Sichuan Agricultural University, Chengdu, China

**Keywords:** *Echinococcus granulosus*, IAPs, anti-apoptosis, infertile cysts, immune evasion

## Abstract

The larval stage of *Echinococcus granulosus* sensu lato, resulting in cystic echinococcosis, a parasitic zoonosis, causes huge economic losses to the livestock industry and poses a threat to public health. Inhibitor of apoptosis proteins (IAPs) is a class of endogenous anti-apoptotic family, which plays a significant functional role in the regulation of organism’s development. Herein, to explore potential functions of IAPs in *E. granulosus*, two members of IAPs from *E. granulosus* (Eg-IAP and Eg-BIRP) were cloned, expressed, and molecularly characterized. Eg-IAP and Eg-BIRP encoded putative 331 and 168 residue proteins, respectively. Bioinformatic analysis showed that both proteins contained a type II BIR domain-the essential functional domain of IAPs. Fluorescence immunohistochemistry revealed that both proteins were ubiquitously localized in all life-cycle stages of *E. granulosus*. Our fluorescent quantitative PCR (RT-qPCR) results revealed relatively higher transcription levels of two Eg-IAPs in protoscoleces (PSCs) compared to the 18-day strobilated worms. We further used different concentrations of LCL161, a Smac-mimetic pan-IAPs inhibitor, to induce the apoptosis in PSCs *in vitro*, and revealed that the survival rate of PSCs and transcription levels of both genes were negatively correlated with the concentration of LCL161. While the results of light microscopy, transmission electron microscopy (TEM), and terminal deoxynucleotidyl transferase dUTP nick end labeling (TUNEL) assay also showed a higher apoptotic rate in PSCs with the increasing concentrations of LCL161. Taken together, our findings provide the reasonable evidence that both Eg-IAP and Eg-BIRP have potential implication in critical anti-apoptotic roles during the development of *E. granulosus*.

## Introduction

The larval stage of *Echinococcus granulosus* (*E. granulosus*) sensu lato is the causative agent of cystic echinococcosis (CE), a parasitic zoonosis with worldwide distribution (Othieno et al., 2018; Pan et al., 2018). It has been estimated that global burden of human CE averages 285,500 disability-adjusted life years (DALYs) (Wen et al., 2019). Besides, the estimated economic losses occurring due to animal echinococcosis are up to $1.2 billion annually, and these losses are linked to the decreased fur quality, hide value, milk production and fecundity in livestock (Wu et al., 2018). At present, the World Health Organization (WHO) has taken CE as one of the 17 neglected tropical diseases (NTDs) targeted for control or eradication by 2050 (Wen et al., 2019).

Dogs and other canids act as the definitive hosts in the life cycle of *E. granulosus*, whereas sheep, goats, cattle, yaks, buffalo or human are recognized as the intermediate hosts (Mcmanus et al., 2012). Adult worms of *E. granulosus*, developing from protoscoleces (PSCs), reside in the small intestines of definitive hosts and release eggs containing oncospheres into the external environment through feces of definitive hosts. Subsequently, these oncospheres migrate to the liver and lungs of intermediate hosts and develop as larvae-hydatid cysts (Yin et al., 2018). The fully developed hydatid cyst (an unilocular fluid-filled bladder) consists of an inner germinal layer supported externally by a tough, elastic, acellular laminated layer, surrounded by a host-produced fibrous adventitial layer. Hydatid cysts could be divided into two types, i.e., fertile cysts and infertile cysts (Caremani et al., 1997; Perdomo et al., 1997; WHO Informal Working Group, 2003). The transmission cycle of *E. granulosus* would progress if definitive hosts ingest the fertile cysts, on which PSCs are found both joined to the germinal layer and free in the hydatid fluid filling the cyst cavity (Paredes et al., 2010; Wang et al., 2018).

Inhibitor of apoptosis proteins (IAPs), a family identified in various species, is the negative regulator of apoptosis (Crook et al., 1993; Mads and Pascal, 2010; Haiying et al., 2011). To date, eight members of human IAPs have been identified. And it has been demonstrated that these IAPs generally contain one to three baculovirus IAP domains (BIRs), and may present a C-terminal really interesting new gene (RING) finger motif or caspase activation recruitment domain (CARD) (Salvesen and Duckett, 2002; Nachmias et al., 2004). BIR, containing an conserved C2HC residue, is the essential functional domain within IAPs. It has been reported that BIR domain could be grouped into type I and type II BIR domain on the basis of the presence or absence of a deep peptide-binding groove (Fraser et al., 1999; Mads and Pascal, 2010). Meanwhile, RING domain, a non-specific IAPs structure, might cause degradation of the caspase by ubiquitination (Lorick et al., 1999; Zhang et al., 2004). Additionally, several IAPs of mammal usually contain a CARD domain predicted to mediate protein-protein interactions (Damgaard and Gyrdhansen, 2011). Intriguingly, IAPs can be antagonized by proteins that inhibit IAP/caspase binding or agents that induce degradation of IAPs. Second mitochondria-derived activator of caspases/direct IAP binding protein with low pI (SMAC/DIABLO), a significant endogenous antagonist of IAPs, inhibits the IAPs-caspase interaction by degrading IAPs and activating the caspases, and therefore triggers the apoptosis (Hird et al., 2015).

It has been reported that purified rSj-BIRP, rSj-IAP, and rSj-cIAP proteins of *Schistosoma japonicum* (*S. japonicum*) may play important roles in parasitic living and development by regulating apoptosis (Peng et al., 2010; Luo et al., 2012; Dao et al., 2014). BIRs of *Caenorhabditis elegans* (C. *elegans*) also seem to inhibit the release of apoptotic body (Fraser et al., 1999). Based on foregoing evidences, it is apparent that IAPs plays a key role in the anti-apoptotic responses in parasites; Nevertheless, to date, molecular and functional characterization of IAPs has not been reported in *E. granulosus*. Therefore, in this study we cloned, expressed, and molecularly characterized two members of IAPs, i.e., Eg-IAP and Eg-BIRP in *E. granulosus*. We also evaluated the localization of Eg-IAP and Eg-BIRP at different stages of *E. granulosus* life cycle and estimated the relative transcription levels of two Eg-IAPs genes between PSCs and 18-day strobilated worms stages. In addition, we evaluated the survival rate, morphological alterations and apoptotic rate in PSCs, and the transcriptional differences of Eg-IAP and Eg-BIRP genes following treatment of PSCs with LCL161, a Smac-mimetic pan-IAPs inhibitor, for inducing the apoptosis *in vitro*.

## Materials and Methods

### Ethics Statement

This study was carried out in accordance with the recommendations of the animal protection law of the People’s Republic of China (a draft animal protection law released on 09/18/2009). The protocol was approved by the Care and Use of Laboratory Animals of the Animal Ethics Committee of Sichuan Agricultural University (Ya’an, China) (Approval No. 2015–028).

### Parasites and Animals

Hydatid cysts were collected from the liver and lungs of naturally infected sheep at an abattoir in Sichuan Province, China. The fertility of cysts was confirmed by observing PSCs within the cysts under the light microscopy. Infertile cysts showed the absence of PSCs by either macroscopic or microscopic observations (Khan et al., 2001). Cyst walls from fertile/infertile cysts and PSCs were separated and treated as previously described (Wang et al., 2018). The 18-day strobilated worms (Supplementary Figure S1) were obtained from the small intestine of an 8-month-old male beagle dog at days 18 post-infection with 50,000 PSCs, and the dog has been euthanized. The genotypes of PSCs, hydatid cysts and 18-day strobilated worms samples were identified as *E. granulosus* G1 strain, and the genes of samples were amplified as reported by Bowles et al. using the JB3/JB4.5 primers (5′-TTTTTTGGGCATCCTGAGGTTTAT-3′/5′-TAAAGAAAGAACATAATGAAAATG-3′) (Bowles et al., 1992). For subsequent experiments, four 9-weeks-old female New Zealand white rabbits were obtained from the Laboratory Animal Center of Sichuan Agricultural University. All animals were provided with food pellets and sterilized water *ad libitum*.

### Bioinformatic Analysis

The cDNA sequences of Eg-IAP (CDS22977.1) and Eg-BIRP (CDS17298.1) were downloaded from NCBI^1^. Basic physicochemical properties of these proteins were predicted using tools available at the ExPaSy website^2^. Secondary structure and functional regions were analyzed using the NPSA_server^3^ and InterProScan^4^. Protein tertiary (3D) structures were downloaded from NCBI. Sequences were aligned, and phylogenetic trees were constructed using MEGA software (version 5.05) using the Maximum-Likelihood (ML) method.

### Cloning, Expression, and Purification of rEg-IAP and rEg-BIRP

Total RNA was extracted from PSCs using the TaKaRa MiniBEST Universal RNA Extraction Kit (TaKaRa, Dalian, China) as per the manufacturer’s guidelines, and the Prime Script^TM^ RT reagent Kit with gDNA Eraser (TaKaRa) was used to synthesize first-strand cDNA. The sequence encoding Eg-IAP was amplified by PCR with primer 5′-CCGGAATTCATGTTCACAGCTTTCCAATC-3′ (including a *Eco*RI site) and primer 5′-CCCAAGCTTCCTAG GCGGAGGTGGA-3′ (including a *Hind*III site). The sequence of Eg-BIRP was amplified using the primers 5′-CCGGAATTC ATGCCGTCACCCTTTCT-3′ (including a *Eco*RI site) and 5′-CCCAAGCTTTTTAACAAGCGAAACCC-3′ (including a *Hind*III site). PCR cycling of Eg-IAP/Eg-BIRP involved an initial denaturation for 5 min at 94°C followed by 30 cycles at 95°C for 45 s, 60/58°C for 45 s, and 72°C for 30 s. The PCR products were ligated into the pET32a (+) plasmid (Invitrogen, Carlsbad, CA, United States), and transformed into *Escherichia coli* BL21 (DE3) competent cells (Cowin Biotech, Beijing, China). Subsequently, the transformants were induced by 1 mM isopropyl β-d-1- thiogalactopyranoside (IPTG) for 8 h, then the bacterial samples were mixed with standard protein loading buffer (5×) (Beyotime, shanghai, China) and heated for 12 min at 95°C. After centrifuged for 5 min at 12,000 rpm, 8 μL supernatant was collected as template to detect the expression of proteins. The recombinant proteins were harvested and purified using Ni^2+^ affinity chromatography (Bio-Rad, Hercules, CA, United States). The expression and purification of proteins were examined by 15% sodium dodecyl sulfate polyacrylamide gel electrophoresis (SDS-PAGE), and the gels were stained with Coommassie blue. Protein concentrations were determined using a BCA protein assay kit (Beyotime, Shanghai, China).

### Serum and Preparation of Polyclonal Antibodies Against rEg-IAP and rEg-BIRP

CE-positive sheep sera was obtained from naturally infected sheep at an abattoir in Sichuan Province, China. And CE-negative sheep sera was obtained from healthy sheep in non-epidemic areas of echinococcosis, and health status was confirmed by autopsy. Four 9-weeks-old female New Zealand white rabbits were immunized four times by subcutaneous injection (2 weeks apart) with rEg-IAP and rEg-BIRP proteins four times, separately. The first immune reagent was 50 μg recombinant protein emulsified with the equal volume of Freund’s complete adjuvant (Sigma, St. Louis, MO, United States), and the booster reagent was 50 μg recombinant protein emulsified with the equal volume of Freund’s incomplete adjuvant. Two weeks after the final immunization, the antiserum was collected, and the serum titer was determined by enzymelinked immuno-sorbent assay (ELISA). Finally, immunoglobulin G (IgG) in the antiserum with high antibody titer was purified using HiTrap Protein A affinity chromatography (Bio-Rad) following the manufacturer’s instructions for subsequent western blotting and immunolocalization. The rabbit serum was obtained and purified before immunization as a negative control as described above.

### Western Blotting

A Mammalian Protein Extraction Kit (Solarbio, Beijing, China) was used to extract total protein from PSCs. Eight microliter purified rEg-IAP/rEg-BIRP proteins and 10 μL of the total protein extracts of PSCs were separated by 15% SDS-PAGE, and transferred to the nitrocellulose membranes (Millipore, Schwalbach, Germany). The membranes were blocked using 5% (*w/v*) skim milk at 37°C for 2 h, then incubated with CE-positive/negative sheep sera, anti-rEg-IAP/anti-rEg-BIRP rabbit IgG, or pre-immunized rabbit sera (1:200 v/v dilution) at 4°C for 12 h, separately. After four washes, membranes were incubated with horseradish peroxidase (HRP)-conjugated sheep anti-rabbit IgG or rabbit anti-sheep IgG (1:2000 v/v dilution, Bio-Rad) for 1 h at 37°C. An Enhanced HRP-DAB Chromogenic Substrate Kit (Tiangen, Beijing, China) was used for detecting the signals.

### Immunolocalization

The samples of PSCs, fertile/infertile cyst walls, and 18-day strobilated worms were fixed with 4% paraformaldehyde, embedded in paraffin wax, and sliced into 5 μm thick sections. Following dewaxing and dehydration, sections were put in 0.01 M citrate buffer, and then 3% H_2_O_2_ was added. After three washes, purified anti-rEg-IAP/anti-rEg-BIRP rabbit IgG as well as native rabbit serum (1:200 v/v dilutions in PBS) were added to the sections and incubated overnight at 4°C. Finally, fluorescein isothiocyanate (FITC)-conjugated sheep anti-rabbit IgG (1:100 dilution in 0.1% Evans blue solution) was added to the specimens and incubated in dark at 37°C for 1 h. The fluorescence signals were observed using a fluorescence microscope (Olympus, Japan), and the images were taken using cellSens at 600 ms exposure time. Negative controls were performed with preimmune rabbit serum.

### Transcriptional Profiles of Eg-IAP and Eg-BIRP at Different Stages of *E. granulosus* Life Cycle

The expression profiles of Eg-IAP/Eg-BIRP genes in PSCs and 18-day strobilated worms stages were analyzed by RT-qPCR using a CFX system (Bio-Rad). Primers specific to Eg-IAP were 5′-A ACACCTCACCACATCTCCTCCTC-3′ and 5′-TCCACAACGC CAGCAGAATCAAG-3′. Primers specific to Eg-BIRP were 5′-G CGGAGCACAAGGCACACTG-3′ and 5′-CGCACCTCCTCCT CGAAGAGATAG-3′. The RNA extraction and cDNA synthesis were performed as described above. PCR cycling of Eg-IAP/Eg-BIRP involved an initial denaturation for 30 s at 95°C followed by 40 cycles at 95°C for 5 s, 62/61°C for 20 s, and 72°C for 30 s. Primers for the *E. granulosus* housekeeping gene beta-actin (*actb*), used as reference gene for normalization, were 5′-ATG GTTGGTATGGGACAAAAGG-3′ and 5′-TTCGTCACAATAC CGTGCTC-3′, and its amplification was performed as described previously (Koskei et al., 2014). Relative expression was calculated using the 2^–ΔΔ*Ct*^ method (Livak and Schmittgen, 2001). Each gene was tested in quadruplicate.

### LCL161-Induced Apoptosis in PSCs *in vitro*

#### *In vitro* Culture and Treatment of PSCs With LCL161

Fresh PSCs at a concentration of 2,500 mL^–1^ were transferred to 6-well microplates, and incubated in an atmosphere containing 5% CO_2_ at 37°C. Each well contained 3 mL of RPMI 1640 medium (Hyclone, Logan, UT, United States), 100 μg⋅mL^–1^ streptomycin, 100 U⋅mL^–1^ penicillin G (Sigma), and 10% fetal calf serum (FCS) (Hyclone).

After 3 days of normal culture, PSCs with viability higher than 95% were divided into five groups. The stock solutions of LCL161 (Canspecsci, Shanghai, China) were added to the culture medium at final concentrations of 200, 400, 800 μmol/L as dosing groups. An equal volume of 0.1% DMSO was added in the culture medium that served as a negative control. The culture medium without LCL161 and DMSO was considered as a blank control. All experiments were performed in triplicate and repeated three times.

#### Survival Rate, Morphological Alterations, and Apoptotic Rate in PSCs Incubated With LCL161 *in vitro*

Following the treatment of PSCs with different concentrations of LCL161 for 8 h, the survival rate of PSCs was assessed by trypan blue staining. And the effects of drug treatment on the trastructural and ultrastructural alterations of PSCs were observed using light microscope and transmission electron microscope (TEM), respectively. For TEM evaluation, PSCs were fixed in 3% glutaraldehyde in 0.1 M PBS for 48 h at 4°C, followed by post-fixing using 2% OsO_4_. Properly fixed PSCs were dehydrated in graded acetones (50–100%) and embedded in Epon Resin (ProSciTech, Australia). Sections (60 nm) were mounted on copper grids, stained with uranyl acetate and lead citrate, and observed using a TEM (Rigaku, Japan) operated at 75 kV.

Additionally, the detection of LCL161-induced apoptosis in PSCs was performed using the Terminal Deoxynucleotidyl Transferase dUTP Nick End Labeling (TUNEL) assay. Five groups of PSCs were collected, respectively, and assays were performed using an *in situ* Cell Death Detection Kit (Roche, Basel, Switzerland) following the manufacturer’s instructions. The detailed TUNEL assay steps were as described previously (Wang et al., 2018). Briefly, PSCs were stained with 1 μg/mL^–1^ of 40,6-diamidino-2-phenylindole (DAPI) reagent (Sigma-Aldrich, St. Louis, MO, United States), and positive cells were confirmed via cells marked with green fluorescence. In TUNEL assay, apoptotic rate (%) was calculated as the percentage of positive cells (the number of positive cells/total cell number ^∗^100).

#### Transcriptional Differences of Eg-IAP/Eg-BIRP in PSCs Incubated With LCL161 *in vitro*

For evaluating the effect of LCL161 treatment on Eg-IAP and Eg-BIRP transcription levels in all five groups of PSCs, RT-qPCR was performed as described above. Relative expression values were calculated using the 2^–ΔΔ*Ct*^ method, and each gene was tested in quadruplicate.

### Statistical Analysis

Data are presented as mean ± standard deviation (SD). For comparison between groups, unpaired Student’s *t*-tests and one-way analysis of variance (ANOVA) were performed using SPSS software (version 22.0), and ^∗^ (*p* < 0.05) were considered statistically significant. All statistical analyses and calculations were performed using GraphPad Prism (Version 7, La Jolla, CA, United States).

## Results

### Bioinformatic Analysis of Eg-IAP and Eg-BIRP

**FIGURE 1:**
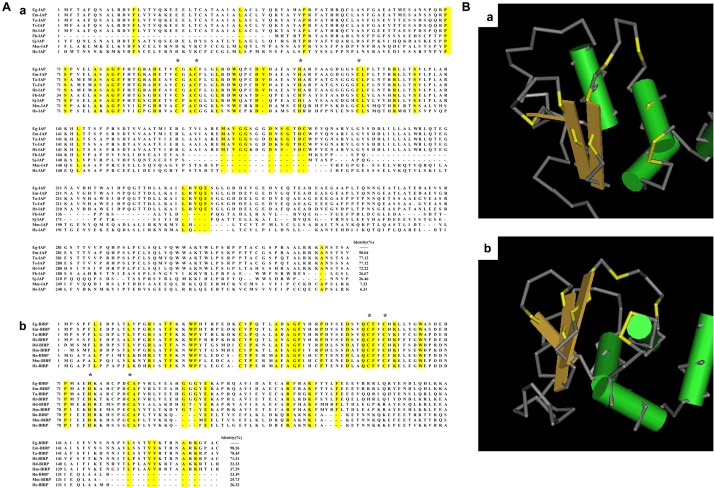
Bioinformatic analysis of Eg-IAP and Eg-BIRP. **(A)** Sequence alignment analysis of Eg-IAP and Eg-BIRP sharing homology with other species. (a) The following sequences of IAP were retrieved from the GenBank: Eg, *Echinococcus granulosus* (Genbank: CDS22977.1); Em, *Echinococcus multilocularis* (Genbank: CDS39460.1); Ta, *Taenia asiatica* (Genbank: VDK35971.1); Ts, *Taenia solium* (Genbank: AQY17500.1); Ht, *Hydatigera taeniaeformis* (Genbank: VDM30371.1); Fh, *Fasciola hepatica* (Genbank: PIS81849.1); Sj, *Schistosoma japonicum* (Genbank: AAW26907.1); Mm, *Mus musculus* (Genbank: AAC53531.1); Hs, *Homo sapiens* (Genbank: AAC50372.1). (b) The following sequences of BIRP were retrieved from the Genbank: Eg, *Echinococcus granulosus* (Genbank: CDS17298.1); Em, *Echinococcus multilocularis* (Genbank: CDI98446.1); Ta, *Taenia asiatica* (Genbank: VDK41268.1); Ht, *Hydatigera taeniaeformis* (Genbank: VDM30835.1); Hd, *Hymenolepis diminuta* (Genbank: VDL61092.1); Hm, *Hymenolepis microstoma* (Genbank: CDS26402.1); Rn, *Rattus norvegicus* (Genbank: NP_071610.1); Mm, *Mus musculus* (Genbank: NP_033819.1); Hs, *Homo sapiens* (Genbank: CAG46540.1). Note, conserved C2HC residue were marked with asterisk. **(B)** 3D structural model of BIR domain in Eg-IAP (a) and Eg-BIRP (b). Predicted peptide-binding groove is colored yellow.

Sequencing analysis showed that full-length Eg-IAP cDNA sequence comprised a 997 bp gene encoding 331 amino acids (aa) with a predicted molecular weight of 35.3 kDa. The Eg-BIRP gene contained an open reading frame (ORF) of 507 bp, encoding a putative protein of 168 aa with a predicted molecular weight of 19.1 kDa. No transmembrane regions or signal peptide sequences were predicted for either IAPs. Subcellular localization analysis demonstrated that both proteins were located in the cytoplasm. Whereas, InterProScan analysis showed that both Eg-IAP and Eg-BIRP contained a BIR functional domain located in aa 65–127, and aa 11–87, respectively. And the conserved C2HC residue of BIR domain was observed in both Eg-IAPs (Figure 1A). In addition, 3D structural analysis revealed that the BIR domain of Eg-IAPs contained a peptide-binding groove, separately (Figure 1B).

Homologous sequence alignment showed that Eg-IAP shared 90.04% identity with IAP from *Echinococcus multilocularis* (*E. multilocularis*), 77.12% with *Taenia solium* (*T. solium*) IAP and *Taenia asiatica* (*T. asiatica*) IAP, followed by 72.22% with *Hydatigera taeniaeformis* (*H. taeniaeformis*) IAP. However, Eg-IAP shared lower identity with sequences of IAPs from mammal, such as *Homo sapiens* (*H. sapiens*) and *Mus musculus* (*M. musculus*). Besides, Eg-BIRP was more similar to equivalent proteins of other cestodes, shared 98.26, 78.45, 71.31% identity with BIRP from *E. multilocularis*, *T. asiatica*, *H. taeniaeformis*, respectively. Similarly, the identity of sequences of BIRP was low between *E. granulosus* and mammal (Figure 1A).

In addition, the ML phylogenetic tree analysis showed that Eg-IAP and Eg-BIRP were located on different branches. Specifically, both Eg-IAPs showed the closest genetic relationship with *E. multilocularis*. However, the mammalian hosts formed another branch, which showed distant genetic relationship with Eg-IAPs (Supplementary Figure S2).

### Expression, Purification, and Western Blotting of rEg-IAP and rEg-BIRP

*Escherichia coli* BL21 (DE3) was induced to express two insoluble rEg-IAPs with a ∼18 kDa His-tag. After purification, the molecular masses of rEg-IAP and rEg-BIRP, yielding a single band, were ∼54 and 36 kDa, respectively, which were close to the expected values. Western blotting showed that both rEg-IAP and rEg-BIRP reacted with CE-positive sheep sera, and with anti-Eg-IAP and anti-Eg-BIRP rabbit sera, respectively. Moreover, anti-Eg-IAP and anti-Eg-BIRP rabbit sera IgGs recognized their respective native proteins in the total protein extracts of PSCs. As expected, no signal was observed when either IAPs were probed with sera from CE-negative sheep or pre-immunized rabbits (Supplementary Figure S3).

### Immunolocalization of Eg-IAP and Eg-BIRP

**FIGURE 2:**
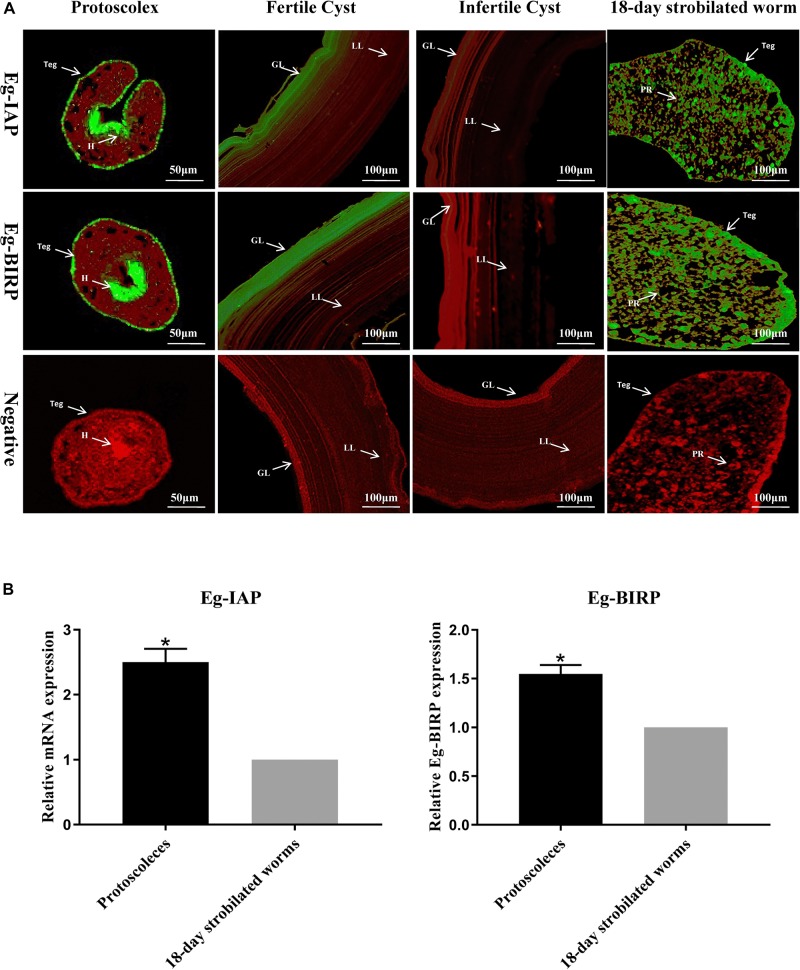
Immunolocalization and Transcription levels of Eg-IAP and Eg-BIRP in *E. granulosus.* **(A)** Fluorescence immunohistochemistry analysis of Eg-IAP and Eg-BIRP at different stages of *E. granulosus* life cycle. rEg-IAP/rEg-BIRP were localized on the protoscolex, fertile/infertile cyst walls, and 18-day strobilated worm using specific anti-rEg-IAP/anti-rEg-BIRP rabbit sera IgG or pre-immune rabbit sera IgG. Abbreviations: H, hooks; LL, laminated layer; GL, germinal layer; Teg, tegument; PR, parenchymal region. Note, Fluorescence-labeled regions were marked with arrows. **(B)** qRT-PCR analysis of transcription levels of Eg-IAP and Eg-BIRP at PSCs and 18-day strobilated worms stages. Data are presented as the means ± SD (*n* = 3). Statistically significant differences between PSCs and 18-day strobilated worms were determined using Student’s *t*-tests (*p < 0.05).

Fluorescence immunohistochemistry analysis showed that both Eg-IAP and Eg-BIRP were mainly distributed in the tegument and hooks of PSCs, the tegument and parenchyma of 18-day strobilated worms, and the germinal layer of fertile cysts. Notably, in the germinal layer of infertile cysts, a weak fluorescence signal of both Eg-IAPs were detected. As expected, no fluorescence was observed with pre-immune rabbit sera IgGs (Figure 2A).

### Transcriptional Profiles of Eg-IAP and Eg-BIRP at Different Stages of *E. granulosus* Life Cycle

The transcription levels of Eg-IAP and Eg-BIRP genes at PSCs and 18-day strobilated worms stages were detected by RT-qPCR. The results showed that transcription levels of both Eg-IAP and Eg-BIRP genes in PSCs were significantly higher (^∗^*p* < 0.05) compared to the strobilated worms stage (Figure 2B).

### Survival Rate, Morphological Alterations, and Apoptotic Rate in PSCs Incubated With LCL161 *in vitro*

**FIGURE 3:**
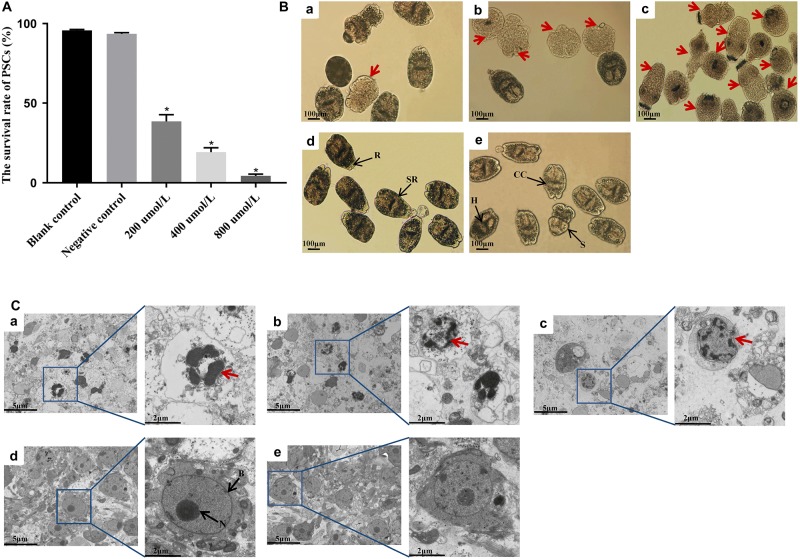
*In vitro* activity of LCL161 against PSCs. **(A)** Survival rate in PSCs incubated with LCL161 for 8 h, and the viability of the PSCs was assessed by Trypan blue staining. Data are presented as the means ± SD (*n* = 3). Statistically significant differences between blank control group and three dosing groups (*p < 0.05), and no significant differences between blank control group and negative control group (*p* > 0.05) were determined using Student’s *t*-tests. Structural alterations **(B)** and ultrastructural alterations **(C)** in PSCs incubated with LCL161 under light microscope and TEM, respectively. (a) PSCs of 200 μmol/L LCL161 group; (b) PSCs of 400 μmol/L LCL161 group; (c) PSCs of 800 μmol/L LCL161 group; (d) PSCs of blank control group; (e) PSCs of negative control group. Abbreviations: B, bilayer; H, hooks; CC, calcareus corpuscles; R, rostellar region; S, sucker; SR, soma region; N, nucleus. Note:Red arrows indicate typical morphological changes associated with apoptosis and black arrows indicate the different structural characteristics of PSCs.

Following the treatment with different concentrations of LCL161 for 8 h, the survival rate of PSCs was shown in Figure 3A. Two control groups of PSCs viability remained above 90% (*p* > 0.05). However, the survival rate of PSCs was reduced to 29.6 ± 3.4% by incubation with 200 μmol/L of LCL161, 19.2 ± 2.8% at 400 μmol/L and 6.7 ± 1.2% at 800 μmol/L, which showed a obvious decrease of viability compared with that of blank control group (^∗^*p* < 0.05).

The results of survival rate tests coincided with the structural and ultrastructural alterations observed by light microscopy and TEM. No morphological changes were observed in the PSCs in the control groups. However, the structure of PSCs in the dosing groups was markedly altered, mainly showing soma contraction, rostellar disorganization, calcareus corpuscles dissolution, and hooks obscure under light microscopy (Figure 3B). In addition, TEM analysis revealed the cellular structure of PSCs in the control groups owned complete bilayer and nuclear membranes, and intact plasmosomes. Nevertheless, typical ultrastructural changes indicative of apoptosis in LCL161-treated PSCs were displayed, including shrinking decreased volume, and irregularly shaped even ruptured nucleus (Figure 3C).

**FIGURE 4:**
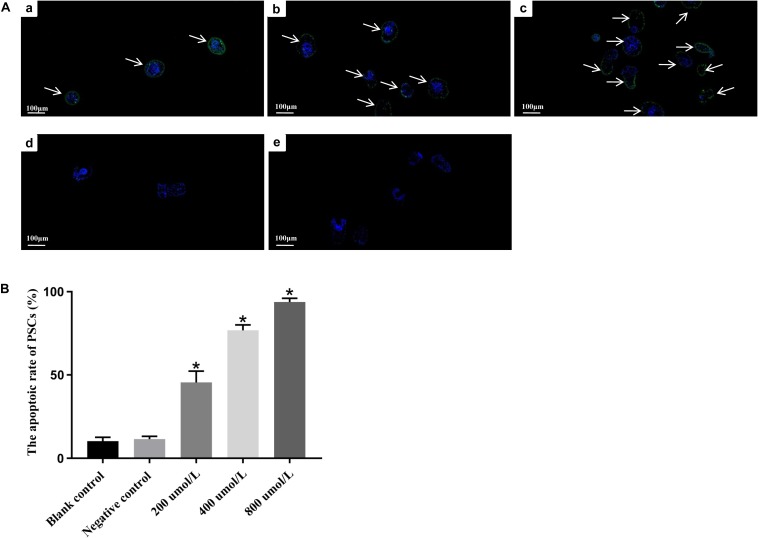
*In vitro* apoptosis of LCL161 against PSCs determined by TUNEL assay. **(A)** Fluorescence signals of apoptotic cells in PSCs incubated with LCL161. (a) PSCs of 200 μmol/L LCL161 group; (b) PSCs of 400 μmol/L LCL161 group; (c) PSCs of 800 μmol/L LCL161 group; (d) PSCs of blank control group; (e) PSCs of negative control group. Note, Fluorescence-labeled regions were marked with arrows. **(B)** The apoptotic rate in PSCs incubated with LCL161 *in vitro* determined by TUNEL assay. Data are presented as the means ± SD (*n* = 3). Statistically significant differences between blank control group and three dosing groups (*p < 0.05), and no significant differences between blank control group and negative control group (*p* > 0.05) were determined using Student’s *t*-tests.

Moreover, TUNEL assay was performed to detect double-stranded breaks in the DNA of cells undergoing programmed cell death. The apoptotic rate of PSCs in blank control group and negative control group were low, i.e., 10.3 ± 2.3% and 11.5 ± 1.7%, respectively. No apparent difference was found between two control groups (*p* > 0.05). However, a significant increase (^∗^*p* < 0.05) in the green fluorescence signals from apoptotic cells was observed in all dosing groups (Figure 4A). As depicted in Figure 4B, the apoptotic rate in PSCs was positively correlated with the concentration of LCL161. The rate of apoptosis in PSCs treated with different concentrations of LCL161 were 45.6 ± 6.7% (200 μmol/L), 76.8 ± 3.2% (400 μmol/L) and 93.8 ± 2.3% (800 μmol/L), respectively.

### Transcriptional Differences of Eg-IAP/Eg-BIRP in PSCs Incubated With LCL161 *in vitro*

**FIGURE 5:**
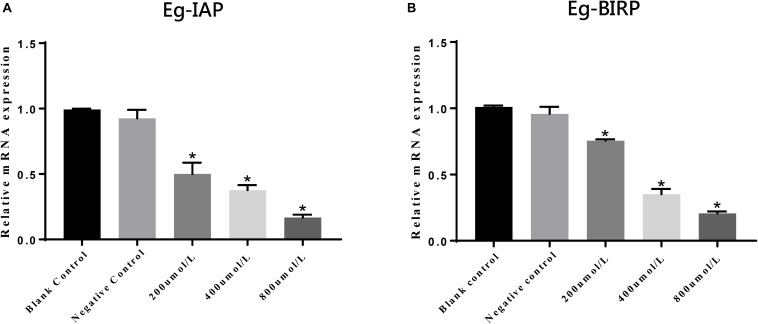
Transcriptional differences of Eg-IAP **(A)** and Eg-BIRP **(B)** in PSCs incubated with LCL161 *in vitro*. Data are presented as the means ± SD (*n* = 3). Statistically significant differences between blank control group and three dosing groups (*p < 0.05), and no significant differences between blank control group and negative control group (*p* > 0.05) were determined using Student’s *t*-tests.

In present study, we also assessed the transcript profiles of Eg-IAP and Eg-BIRP genes following incubation of PSCs with different concentrations of LCL161. Briefly, our RT-qPCR results showed that the transcription levels of both Eg-IAP and Eg-BIRP were significantly (^∗^*P* < 0.05) down-regulated, and this change in expression was more pronounced when PSCs were treated with the increasing concentrations of LCL161 (Figure 5).

## Discussion

At present, it is well established that apoptotic and anti-apoptotic mechanisms are the essential biological features in a variety of species. Apoptotic mechanisms play an important role in removing the metabolites in parasites including cestodes, nematodes and trematodes (Orme and Meier, 2009). Indeed, whether loss of apoptotic regulation or excess apoptosis might result in a series of diseases (Mattson, 2000; Rathmell and Thompson, 2002). On the other hand, halting apoptosis inevitably lead to inability to eradicate tumor cells in anti-cancer therapy (Reed, 2000; Nachmias et al., 2004). Accordingly, tight regulation of apoptosis is necessary for maintaining the tissue homeostasis, even a delicate balance of life and death. In the last decade, a complex network of pro- and anti-apoptotic proteins was revealed, of which members of IAPs family, playing an important role in suppression apoptotic signals, are the special mention and require due consideration (Vaux and Strasser, 1996; Dosreis and Barcinski, 2001; Orme and Meier, 2009; Prado et al., 2015).

IAPs was initially discoved in the baculoviral genome, and identified in various organisms ranging from yeast, nematodes, flies, and human since then (Nachmias et al., 2004). In this study, we preliminarily studied Eg-IAP and Eg-BIRP proteins from the perspective of molecular and functional characterization. Similar to Sj-IAP and Sj-BIRP, molecular characterization revealed that both Eg-IAP and Eg-BIRP proteins characterized herein contained no RING and CARD domain, and only showed a single BIR domain of ∼70 aa residue, which is consistent with the length reported in previous reports (Peng et al., 2010; Dao et al., 2014). BIR domain, the defining feature of IAPs, is protein interacting modules with specific distinct binding properties (Eckelman et al., 2008). It has been reported that Type I BIR domain, lacking the groove, is incapable of binding caspases or IAP antagonists but use distinct modes to interact with their target proteins, such as tumor necrosis factor receptor (TNFR)-associated factor 1 (TRAF1) and TRAF2, transforming growth factor-β (TgFβ) activatedkinase (TAK1) binding protein, TAB1 (Rothe et al., 1995; Uren et al., 1996; Lu et al., 2007). Nevertheless, the peptide-binding specificity of Type II BIR domains makes it could mediate protein-protein interaction with partners that contain an IAP-binding motif (IBM), which has been described in caspases and Smac/DIABLO. Intriguingly, in this study, the presence of peptide binding groove proved BIR domain of both Eg-IAPs belonged to Type II, confirmed theoretically that Eg-IAP and Eg-BIRP possessed the functional attributes required for inhibiting apoptosis via binding the IBM of caspase (Tamm et al., 1998; Salvesen and Duckett, 2002). As for CARD domain, it has been reported that CARD could inhibit activation of cIAP1’s E3 activity, and suppress cell proliferation and migration in mammal. Further, CARD is also necessary to maximally suppress caspase-dependent apoptosis (Lopez et al., 2011). But it seems different from mammal, no CARD domain has been found in helminth including *E. granulosus* so far, which implied CARD may be not the part of the species’s apoptotic process. In addition, the analysis of ML phylogenetic tree showed that both Eg-IAPs shared low homology with the IAPs from mammalian hosts, suggesting that Eg-IAPs may have species-specific functions in *E. granulosus*.

Generally, the host’s immune system attacks the invading parasites by triggering the apoptosis of invaders for self-protection (Prado et al., 2015). Conversely, previous studies have indicated that parasitic helminths could evade the vigorous immune responses in hosts through the anti-apoptotic pathways and related factors (Giri and Roy, 2016). It has been reported that long-term survival of *S. japonicum* in hosts is mainly attributed to the anti-apoptotic function of IAPs family (Heussler et al., 2001; Verhagen et al., 2001; Liu et al., 2018). Similarly, despite facing the host immune response, the adult worms of *E. granulosus* could parasitize in the small intestines of definitive hosts for nearly 6 months, and the larvae can even survive for several decades in the liver and lungs of intermediate hosts. *E. granulosus* is structurally like a flipped gut, its tegument not only is an important organ for nutrient absorption and waste excretion, also plays a key role in escaping the attack from host immune system. In this study, the results of immunolocalization assay revealed that both Eg-IAP and Eg-BIRP were widely distributed in the germinal layer of hydatid cysts and the tegument of PSCs and 18-day strobilated worms. These findings suggested that Eg-IAPs might be involved in combating and attenuating the host immunological responses in *E. granulosus*. Notably, as shown by RT-qPCR, the transcription levels of Eg-IAP and Eg-BIRP genes were higher in PSCs compared to the strobilated worms stage. Interestingly, the expression levels of Eg-IAPs in this study were consistent with that in the transcriptomic database for *E. granulosus* reported by Zheng et al. (2013). Accordingly, we hypothesized that the Eg-IAPs may play a more critical role in the growth and development of PSCs.

It is noteworthy that the fluorescence intensities of Eg-IAP and Eg-BIRP were weaker in the germinal layer of infertile cysts compared with germinal layer of fertile cysts. The formation of infertile cyst is a special biological phenomenon of *E. granulosus.* In infertile cysts, caspase 3 enzyme activity, apoptosis-inducing ligands and DNA fragmentation are increased relative to fertile cysts (Wang et al., 2019). Evidence suggests that apoptosis negatively regulates the PSCs development and causes infertility in hydatid cysts, but the genes involved in regulating the process still unknown (Gonzalo et al., 2010; Paredes et al., 2010; Spotin, 2012). Therefore, it would be reasonable to believe that Eg-IAPs, as an anti-apoptotic warrior, has the irreplaceable ability to maintain the fertility of hydatid cysts, but further study should be carried out to verify this assumption.

At present, siRNA-mediated knockdown of IAPs and its small molecule inhibitors are widely used in functional studies of this family, and the application of both assays is very effective (Dai et al., 2009; Yang et al., 2016; Liu et al., 2018). Among the small molecule inhibitors of IAPs, Smac/DIABLO, selectively binds to the type II BIR domains, is considered to be the best characterized antagonist of IAPs that can increase apoptosis. And Smac mimetics are artificially synthesized small-molecule compounds that mimic the apoptotic function of Smac/DIABLO protein (Hird et al., 2015; Fulda, 2017). In this study, we used the pan IAPs inhibitor-LCL161 drug, a Smac mimetics (Infante et al., 2014; Tian et al., 2014; Najem et al., 2016), to induce the apoptosis in PSCs *in vitro*. LCL161 has been tested as an anticancer agent in phase I and phase II clinical trials, and the anti-proliferative effect of the drug was confirmed in some solid tumors, such as non-small cell lung cancer (Yang et al., 2016), Hepatocellular carcinoma (HCC) (Tian et al., 2014), and leukemia (Weisberg et al., 2011), et al. In order to observe a significant effect in a short time, the high doses of LCL161 were administered in the present study. As shown in Figure 3, with the increasing concentrations of LCL161, the morphological changes indicative of apoptosis in PSCs was more obvious, the apoptotic rate in PSCs was gradually up-regulated, and the transcription levels of both IAP and BIRP genes were also significantly down-regulated. Garside (Garside et al., 2010) reported that in helminth infections, caspase, as a TNFα-related factor, can induce the apoptosis in parasites through external signals to hinder their growth. Intriguingly, results of present study also demonstrated that, LCL161-mediated degradation/disruption of the IAPs in caspase-IAPs complexes might have triggered the activity of caspase and result the apoptosis in PSCs, and further studies will be carried out to evaluate the mechanism of Eg-IAPs inhibiting caspase activity in *E. granulosus*. The results of this *in vitro* experiment also provide a reasonable evidence that Eg-IAP and Eg-BIRP might have the essential roles in modulating the anti-apoptotic responses during the development of *E. granulosus*.

## Conclusion

To sum-up, both Eg-IAP and Eg-BIRP contained a type II BIR domain, distributed in all life-cycle stage of *E. granulosus*, and showed higher transcription levels in PSCs compared to the 18-day strobilated worms stage. In addition, with the increasing concentrations of pan IAPs inhibitor-LCL161, the survival rate of PSCs and transcription levels of Eg-IAP and Eg-BIRP genes gradually decreased, whereas the apoptotic rate in PSCs was relatively up-regulated. Therefore, we envisaged that Eg-IAP and Eg-BIRP are actively implicated in the modulation of anti-apoptotic mechanisms during the development of *E. granulosus*, which may play a important role in maintain the fertility of hydatid cysts, and could be related to the immune evasion of *E. granulosus*.

## Data Availability Statement

All datasets generated and analyzed for this study are included in the article/Supplementary Material.

## Ethics Statement

The animal study was reviewed and approved by the Care and Use of Laboratory Animals of the Animal Ethics Committee of Sichuan Agricultural University (Ya’an, China) (Approval No. 2015–028).

## Author Contributions

JZ and GY conceived the study. JZ and HS designed the study and wrote the first version of the manuscript. NW, CG, NS, CA, RH, and YS participated in study design and coordination, and performed statistical analyses. XG and WL conceived the study and collected experimental material. YX and XP collected and analyzed the raw data. CA helped in discussion and revision of initial draft of manuscript. GY was responsible for the study, participated in its design and coordination, and helped to draft the manuscript. All authors read and approved the final manuscript.

## Conflict of Interest

The authors declare that the research was conducted in the absence of any commercial or financial relationships that could be construed as a potential conflict of interest.

## Footnotes

1 https://www.ncbi.nlm.nih.gov/

2 http://web.expasy.org/protparam/

3 http://npsa-pbil.ibcp.fr/

4 http://www.ebi.ac.uk/InterProScan/

## Supplementary Material

FIGURE S1 Structure of 18-day strobilated worm under light microscope. Images of strobilated worm were magnified ×400. B, band; CC, calcareus corpuscles; EC, excretory canal; GR, genital rudiment; H, Hooks; R, rostellum; S, sucker; SG, segment.

FIGURE S2 Phylogenetic tree of Eg-IAP and Eg-BIRP using the Maximum- Likelihood (ML) method. GenBank accession numbers are shown after the species name.

FIGURE S3 Expression, purification (Lane 1–3) and western blotting (Lane 4–8) of rEg-IAP **(A)** and rEg-BIRP **(B)**. M, Molecular mass markers (kDa); lane 1, pET32a(+)-vector; lane 2, pET32a(+)-Eg-IAP/pET32a(+)-Eg-BIRP; lane 3, Purified rEg-IAP/rEg-BIRP; lane 4, Purified rEg-IAP/rEg-BIRP probed with anti-rEg-IAP/anti-rEg-BIRP rabbit sera IgG; lane 5, Purified rEg-IAP/rEg-BIRP probed with pre-immunized rabbit sera IgG; lane 6, Purified rEg-IAP/rEg-BIRP probed with sera from CE-positive sheep sera; lane 7, Purified rEg-IAP/rEg-BIRP probed with CE-negative sheep sera; lane 8, Total protein extracts of PSCs probed with anti-rEg-IAP/anti-rEg-BIRP rabbit sera IgG.
