# Supplementary figures and images for: Molecular and Functional Characterization of Inhibitor of Apoptosis Proteins (IAP, BIRP) in Echinococcus granulosus
Source: Front Microbiol. 2020 Apr 22;11:729. doi: 10.3389/fmicb.2020.00729 (PMC7188921; doi:10.3389/fmicb.2020.00729)

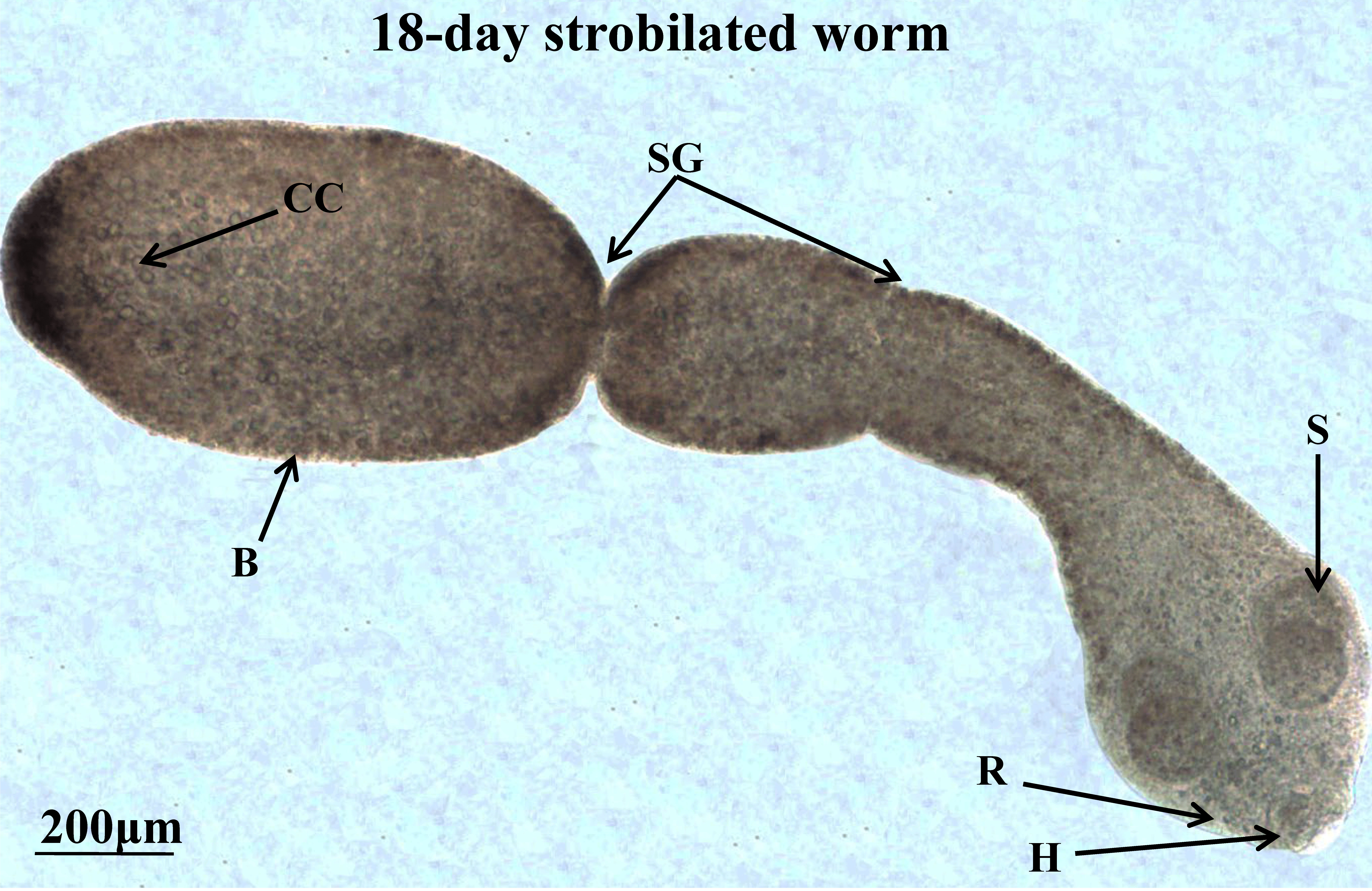

Supplement: FIGURE S1 — Structure of 18-day strobilated worm under light microscope. Images of strobilated worm were magnified ×400. B, band; CC, calcareus corpuscles; EC, excretory canal; GR, genital rudiment; H, Hooks; R, rostellum; S, sucker; SG, segment. [file Image_1.tiff]

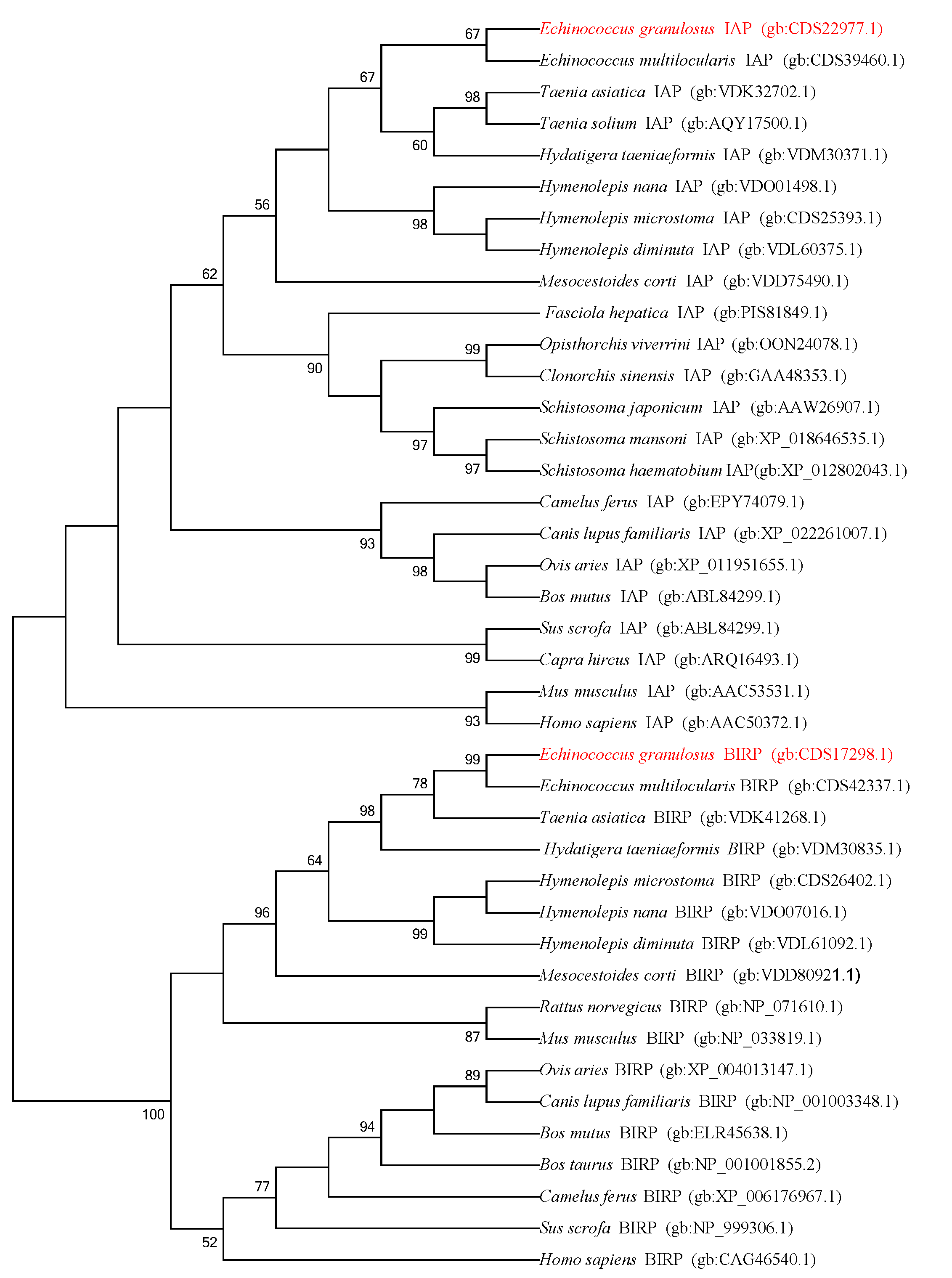

Supplement: FIGURE S2 — Phylogenetic tree of Eg-IAP and Eg-BIRP using the Maximum- Likelihood (ML) method. GenBank accession numbers are shown after the species name. [file Image_2.tiff]

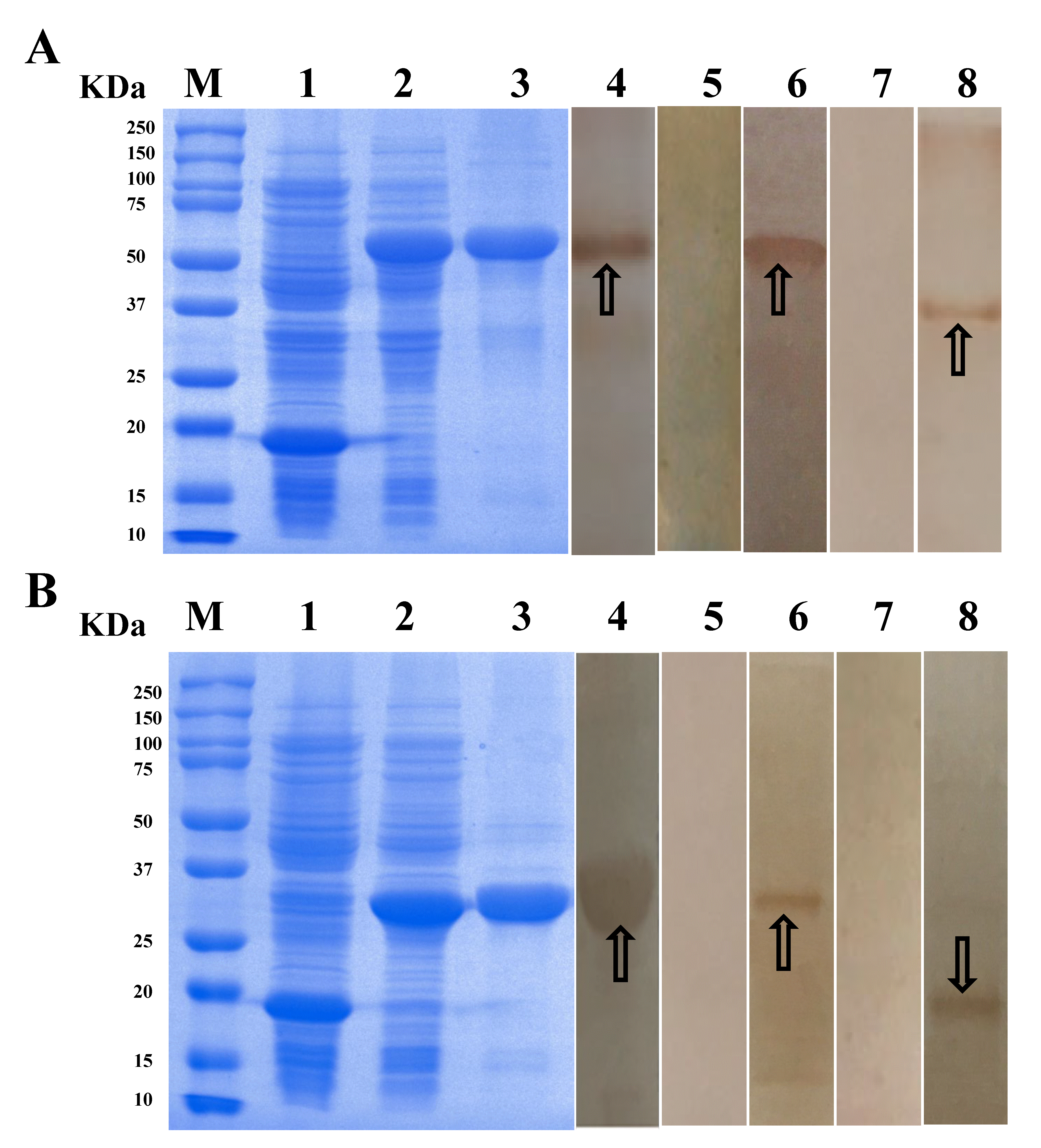

Supplement: FIGURE S3 — Expression, purification (Lane 1–3) and western blotting (Lane 4–8) of rEg-IAP (A) and rEg-BIRP (B). M, Molecular mass markers (kDa); lane 1, pET32a(+)-vector; lane 2, pET32a(+)-Eg-IAP/pET32a(+)-Eg-BIRP; lane 3, Purified rEg-IAP/rEg-BIRP; lane 4, Purified rEg-IAP/rEg-BIRP probed with anti-rEg-IAP/anti-rEg-BIRP rabbit sera IgG; lane 5, Purified rEg-IAP/rEg-BIRP probed with pre-immunized rabbit sera IgG; lane 6, Purified rEg-IAP/rEg-BIRP probed with sera from CE-positive sheep sera; lane 7, Purified rEg-IAP/rEg-BIRP probed with CE-negative sheep sera; lane 8, Total protein extracts of PSCs probed with anti-rEg-IAP/anti-rEg-BIRP rabbit sera IgG. [file Image_3.tiff]
